# Efficacy of Degarelix in the Neoadjuvant Setting in Managing Locally Advanced Prostate Cancer

**DOI:** 10.7759/cureus.40752

**Published:** 2023-06-21

**Authors:** Sindhu Sankaran, Thirumalai Ganesan Govindaswamy, Kunal Dholakia, Nivash Selvaraj, Deerush Kanan, Madhav Tiwari, Narasimhan Ragavan

**Affiliations:** 1 Urology, Apollo Hospitals, Chennai, IND; 2 Urology, Apollo Hospitals, Chennai , IND

**Keywords:** androgen deprivation therapy, locally advanced prostate cancer, radical prostatectomy, neoadjuvant therapy, degarelix

## Abstract

Background

Prostate cancer holds a substantial presence in the global cancer landscape, and a considerable proportion of diagnoses occur at late stages, particularly in India. Management of locally advanced prostate cancer necessitates a multimodal treatment strategy. A critical part of this strategy is neoadjuvant androgen deprivation therapy, typically administered via luteinizing hormone-releasing hormone (LHRH) analogs. This study explores the potential of an alternative approach: neoadjuvant therapy with degarelix, an LHRH antagonist, and its impact on perioperative and postoperative outcomes in patients undergoing radical prostatectomy for locally advanced or high-risk prostate cancer.

Methodology

We conducted a retrospective, non-randomized clinical study at Apollo Hospitals in Chennai, India. Patients diagnosed with locally advanced or high-risk prostate cancer who underwent radical prostatectomy were included. Participants were patients treated with neoadjuvant degarelix and subsequent radical prostatectomy between March 2020 and June 2022. We excluded patients receiving radical radiotherapy, those switching from LHRH agonists to antagonists, and those contraindicated for androgen deprivation therapy due to existing comorbidities. For comparison, we selected a group from the institutional database who received conventional treatment (i.e., without neoadjuvant therapy).

Results

The study compared two groups, each with 32 patients. The groups had no significant difference in total operative duration and console times. The postoperative pathological assessment showed significantly lower margin positivity rates and notable pathological downstaging in the group receiving neoadjuvant degarelix compared to the control group. The incidence of node positivity, prostate-specific antigen levels at three months postoperative, and number of pads used per day at one month did not differ significantly between the two groups.

Conclusions

Our study suggests that neoadjuvant degarelix could notably enhance patient outcomes in locally advanced prostate cancer management. The benefits include improved symptom control, significant reductions in margin positivity rates, and facilitated surgical procedures. Neoadjuvant degarelix therapy could potentially enhance the feasibility of the surgical intervention in locally advanced prostate cancer management, thus suggesting a promising pathway for improved patient care.

## Introduction

Prostate cancer ranks as the fourth most prevalent cancer and the second most frequent cause of cancer-related death in men worldwide [1]. The incidence is on the rise in India, with a startling 85% of diagnoses occurring at stages III and IV, as opposed to early-stage identification common in Western countries [2].

Locally advanced prostate cancer typically refers to a T3 or T4 diagnosis determined through clinical or radiological examination. This condition is invariably high-risk and may present with additional risk factors such as enlarged nodes, prostate-specific antigen (PSA) values over 20 ng/ml, or a Gleason sum exceeding seven. Managing locally advanced prostate cancer necessitates a multimodal approach, one facet of which is androgen deprivation therapy, typically employing either luteinizing hormone-releasing hormone (LHRH) agonists or antagonists [3]. LHRH agonists, used in the neoadjuvant setting, can enhance pathological outcomes by reducing the tumor size, decreasing positive surgical margin rates, and promoting tumor downstaging [4,5]. However, they have not yet proven effective in enhancing overall patient survival [5].

Introduced in 2008, the LHRH antagonist degarelix has emerged as an innovative multimodal therapy option [6]. Its use in advanced prostate carcinoma cases has considerably improved lower urinary tract symptoms and enhanced patient-related quality of life compared to LHRH agonists [7,8]. Despite a few studies examining its use in a neoadjuvant setting before external beam radiation for intermediate and advanced prostate cancers, degarelix has not been explored in a neoadjuvant context preceding radical prostatectomy, particularly in cases of locally advanced prostate cancers [7-10]. Therefore, we conducted this study to evaluate the impact of neoadjuvant degarelix on the perioperative and postoperative outcomes of patients undergoing radical prostatectomy for locally advanced or high-risk prostate cancer.

## Materials and methods

We performed a retrospective, non-randomized clinical study at Apollo Hospitals, Chennai, India, focused on patients with locally advanced or high-risk prostate cancer undergoing radical prostatectomy as part of multimodal therapy. The study encompassed patients diagnosed with locally advanced or high-risk prostate cancer who underwent treatment with neoadjuvant degarelix followed by radical prostatectomy between March 2020 and June 2022. Degarelix was administered at the standard dose of 240 mg loading dose (as two 120 mg subcutaneous injections) and subsequently monthly 80 mg subcutaneous injections. We excluded patients treated with radical radiotherapy, those who transitioned their neoadjuvant therapy from LHRH agonists to LHRH antagonists, and individuals contraindicated for androgen deprivation therapy due to existing medical comorbidities. For comparison purposes, we selected a similar patient population treated in the standard manner (without neoadjuvant therapy) from the institutional database. The study received approval from the institutional ethics committee (Reference No: AMH-C-S-057/11-22).

We gathered demographic information, cancer parameters, perioperative and postoperative surgical parameters, pathological outcomes, continence, erectile function, three-month postoperative PSA, and adjuvant therapy details from the identified patient population.

Data analysis involved calculating the mean and standard deviation for continuous variables and percentages for discrete variables. Parametric tests compared the two groups, depending on the variable type. We deemed differences statistically significant if the p-value was less than 0.05.

## Results

We compared two patient groups: Group A, which received neoadjuvant degarelix (n=32), and Group B, with similar disease characteristics, chosen from the institutional database (n=32). Both groups were comparable in terms of mean ages. The average number of monthly neoadjuvant degarelix doses administered was 2.91. The mean preoperative PSA and the number of individuals with preoperative histopathology across different Gleason grade groups remained consistent between both populations (Table 1).

**Table 1:** Patient demographics and preoperative parameters SD, standard deviation; LUTS, lower urinary tract symptoms; PSA, prostate-specific antigen; GG, Gleason group

Parameter	Group A (Neoadjuvant degarelix)	Group B (No neoadjuvant degarelix)	P-value
Age mean±SD years	66.906±7.74	65.34±8.32	0.2235
BMI mean±SD kg/m^2^	24.72±3.08	24.77±3.88	0.5
Presence of LUTS preoperatively (%)	81.25	65.62	0.077
Preoperative PSA mean ± SD mg/dl	44.490±55.16	33.16±44.22	0.240
Gleason score 6 (GG1, %)	3.125	13.33	0.176
Gleason score 7 (GG2, %)	37.5	46.66	
Gleason score 8, 9, 10 (GG 4 and 5, %)	59.375	40	

Total operative duration and console times were similar for both groups (Group A: 224.6 minutes vs. Group B: 228.5 minutes, p=0.4; and Group A: 178.4 minutes vs. Group B: 180.5 minutes, p=0.2). Neither group experienced intraoperative or postoperative complications, and no patients required transfusions (Table 2).

**Table 2:** Intraoperative details SD, standard deviation; NA, not applicable

Parameter	Group A (Neoadjuvant degarelix)	Group B (No neoadjuvant degarelix)	P-value
Total operative time (Mean±SD minutes)	224.66±29.97	228.59±42.85	0.408
Total console time (Mean±SD minutes)	178.4±36.04	180.53±30.81	0.22
Incidence of intraoperative complications (%)	0	0	NA
Conversion to open surgery (%)	0	0	
Requirement of blood transfusion (%)	0	0	
Length of hospital stay (Mean ± SD days)	1.56±1.27	1.4±0.89	0.558

Upon postoperative pathological examination, significant differences emerged between the groups. Patients in the neoadjuvant group exhibited lower margin positivity rates (34.4% vs. 64.28%; p=0.02). We also observed that a significant number of neoadjuvant degarelix patients underwent pathological downstaging (relative to their pretreatment radiological findings) compared to those who did not receive degarelix (Table 3; p=0.04). No significant differences emerged in the incidence of node positivity, postoperative PSA at three months, and the number of pads used per day at one month between the two groups. All patients in both groups achieved continence recovery (one pad a day or less) by three months.

**Table 3:** Postoperative data SD, standard deviation; HPE, histopathological evidence; PSA, prostate-specific antigen

Parameter	Group A (Neoadjuvant degarelix)	Group B (No neoadjuvant degarelix)	P-value
Incidence of multifocal margin positivity HPE (%)	34.4	64.28	0.02
Pretreatment radiological and postoperative pathological T stage of disease (static, %)	59.375	65.625	0.04
Pretreatment radiological and postoperative pathological T stage of disease (worsening, %)	9.375	25	
Pretreatment radiological and postoperative pathological T stage of disease (improvement, %)	31.25	9.375	
Node Yield mean±SD	13.41±4.67	14.23±4.47	0.726
Number of positive nodes mean±SD	0.615±1.00	1.33±2.3	0.4
Node positivity (%)	31.25	25	0.57
Postoperative PSA at 3 months median±SD	0.038±0.03	0.035±0.05	0.407

## Discussion

Diagnosis of prostate cancer involves a multi-faceted approach incorporating PSA levels, positive imaging findings (e.g., multiparametric magnetic resonance imaging (MRI), prostate-specific membrane antigen (PSMA) positron emission tomography (PET) scan), and histopathological confirmation via either transperineal or transrectal biopsy of the prostate gland. This diagnostic process confirms the disease and assists surgeons in risk stratification, thus facilitating treatment planning [3].

Treatment of locally advanced prostate cancers demands a multimodal approach, encompassing options such as androgen deprivation therapy, radical prostatectomy, radiotherapy, and newer hormonal therapies like abiraterone acetate and enzalutamide [3]. While neoadjuvant androgen deprivation therapy with LHRH agonists like goserelin and leuprolide has been explored, the use of neoadjuvant degarelix prior to radical prostatectomy has yet to be clinically tested to comprehend its role within the multimodal therapy paradigm [7].

Degarelix, as opposed to LHRH agonists, immediately induces castration levels of testosterone without an initial surge. Klotz et al. demonstrated that 95% of patients achieved testosterone suppression by the third day following a 240 mg injection of degarelix [6]. This swift onset of action proves beneficial in cases of advanced prostate cancer, where symptoms such as bladder outflow obstruction, ureteric obstruction, or spinal cord compression require urgent relief. A randomized trial by Mason et al. found that neoadjuvant use of degarelix before radiotherapy notably improved patient urinary symptoms compared to goserelin [9]. Hata et al. also revealed that LHRH antagonists significantly mitigated acute urinary toxicities during radiation therapy [11]. In our study, all patients who received degarelix reported improvements in various symptoms, including lower urinary tract symptoms, lower back pain, and easy fatigability (subjective and hence not recorded on a formal measurement scale).

Follow-up examination of all patients who received degarelix indicated a clinical reduction in prostate size and disease extent. PSMA, PET, and MRI results also demonstrated decreased prostate size in patients who underwent pre-operative radiological assessment post-neoadjuvant therapy. While the operative and console times did not significantly differ statistically between the two groups, surgeons subjectively reported increased ease of operability in the degarelix group compared to the non-degarelix group. A better definition of the plane between the prostate and rectum was noted in Group A patients compared to their initial imaging findings at diagnosis. Patients receiving neoadjuvant degarelix demonstrated a significant reduction in positive surgical margins and disease downstaging, suggesting the potential of neoadjuvant degarelix to enhance operative outcomes in patients with locally advanced prostate cancer.

This study is not without limitations, one of which is its non-randomized comparison. Androgen deprivation therapy in the neoadjuvant setting has not increased PSA relapse-free survival [3,7]. However, we believe that long-term follow-up studies incorporating other modalities, such as short-term degarelix, radiotherapy, and immune checkpoint modulators as adjuvant therapies, might show an improvement in the overall survival of patients with locally advanced prostate cancers, thereby reinforcing the role of surgery in the treatment algorithm for these patients.

## Conclusions

Our study indicates that neoadjuvant therapy with degarelix, an LHRH antagonist, could significantly enhance patient outcomes in managing locally advanced prostate cancer. Using neoadjuvant degarelix may improve symptom control, significantly reduce margin positivity rates, and increase surgical ease. Additionally, neoadjuvant degarelix potentially makes surgical intervention a more viable option in locally advanced prostate cancer, indicating a potential pathway for enhanced management of prostate cancer. However, while these results are promising, it is important to note that this was a non-randomized study. Further confirmation from randomized control trials is necessary to substantiate these findings.
